# Craniofacial shape in patients with beta thalassaemia: a geometric morphometric analysis

**DOI:** 10.1038/s41598-020-80234-z

**Published:** 2021-01-18

**Authors:** Petros Roussos, Anastasia Mitsea, Demetrios Halazonetis, Iosif Sifakakis

**Affiliations:** 1 Department of Orthodontics, School of Dentistry, National and Kapodistrian University of Athens, 2, Thivon Str., 11527 Athens, Greece; 2 Department of Oral Diagnosis and Radiology, School of Dentistry, National and Kapodistrian University of Athens, Athens, Greece

**Keywords:** Anatomy, Medical research

## Abstract

The shape of the craniofacial complex of patients with beta thalassaemia was evaluated using geometric morphometrics on lateral cephalometric radiographs and was compared with matched controls. The beta thalassaemia group consisted of 40 patients (16 females, 24 males, mean age 33.4). Each patient was matched by age and gender to two controls (32 females, 48 males, mean age 33.1). The 120 lateral cephalometric radiographs were digitized and traced with 15 curves, 10 landmarks and 117 sliding semi-landmarks. These landmarks were subjected to Procrustes superimposition and principal component analysis in order to describe shape variability of the cranial base, maxilla and mandible, as well as of the entire craniofacial complex for each sex. The first 4 principal components accounted for 50% of the total sample’s variability. The beta thalassaemia group was significantly different in overall shape to the control group for both sexes. Similar findings were noted for the maxilla, the mandible and the cranial base. The main differences were related to smaller mandibular body for the thalassaemia group, midface protrusion and decrease in posterior face height. The shape of the craniofacial complex in these patients is prone to be more convex and hyperdivergent.

## Introduction

Thalassaemia syndromes are hereditary haemolytic anaemias characterised by absent or reduced synthesis of one or more globin chains of haemoglobin. The decline in globin chain production leads to decreased assembly of functioning haemoglobin tetramers resulting in unpaired α and β chains that are incapable of properly releasing oxygen^1–6^. The accumulation of unpaired chains disrupts the majority of the organ systems, leading to high mortality rate, if left untreated^2,6–8^.

Thalassaemias are divided into alpha and beta, according to the affected gene. Beta thalassaemias are classified as major, intermediate and minor, depending on the level of β chain production^1^. Clinical manifestations differ, varying from minor morphologic abnormalities to life threatening conditions^2–12^. Skeletal changes are produced by the hypertrophy of erythroid marrow^7^, resulting in expansion of marrow cavities and consequent skeletal deformities, osteoporosis and pathological fractures^13^. The cranial vault, the maxilla and the vertebrae are the craniofacial structures most commonly affected leading to a convex face. Controversy is reported in the literature as some studies report protrusion of the frontal bones and the cheekbones, depression of the bridge of the nose and cant of the eyes^1,11^, while a recent anthropometric study resulted in no statistical differences of the head and the eye regions between thalassaemia patients and healthy controls^7^. Tooth size and dental arch dimensions are often reduced, the development and eruption of dentition is delayed and tooth discolouration is routinely apparent^8^. The dentition is often displaced by the marrow expansion resulting in interdental spacing and flared maxillary incisors, Class II intermaxillary relationship, increased overjet and anterior open‐bite^8^.

Previous studies on the structure of the craniofacial complex of beta thalassaemia patients relied on clinical examination or conventional cephalometric analysis^10,11,13,15–18^. The latter has well-known inherent limitations. A major problem is its reliance on a few landmarks that serve as a reference basis for the measurements. Point Nasion, at the nasofrontal suture, and point Orbitale, at the inferior orbital rim, are commonly used. Both these points are potentially affected by the skeletal manifestations of beta thalassaemia, so any cephalometric measurements based on them are difficult to interpret and potentially unreliable.

Geometric morphometrics (GM) offers another perspective for studying shape variation, circumventing some of the deficiencies of conventional cephalometrics^19–22^. Combined with modern statistical analysis methods, it offers the possibility of comparing the craniofacial pattern between two subjects or groups, invariant to location, scale and orientation^19–21^. GM methods have been used to study shape differences between healthy controls and patients affected by major syndromes, such as Down’s syndrome^23^, fetal alcohol syndrome^24^, Glut-1Ds^25^, 22q11.2 deletion syndrome^26^, Apert’s syndrome^27^, Pierre Robin sequence and Treacher Collins syndrome^28^, as well as syndromic and nonsyndromic craniosynostoses^29^.

The present study considered three main advantages of GM, compared to traditional cephalometrics, for the evaluation of patients with thalassaemia; (a) there is no strong reliance on a reference structure, such as the anterior cranial base, which may also be affected by the condition, (b) sliding semilandmarks are available for a much denser description of the anatomy, including the curves between customary landmarks, and, therefore, (c) sub-regions of the craniofacial complex can be independently studied, even when the number of conventional landmarks on them is small.

**Figure 1:**
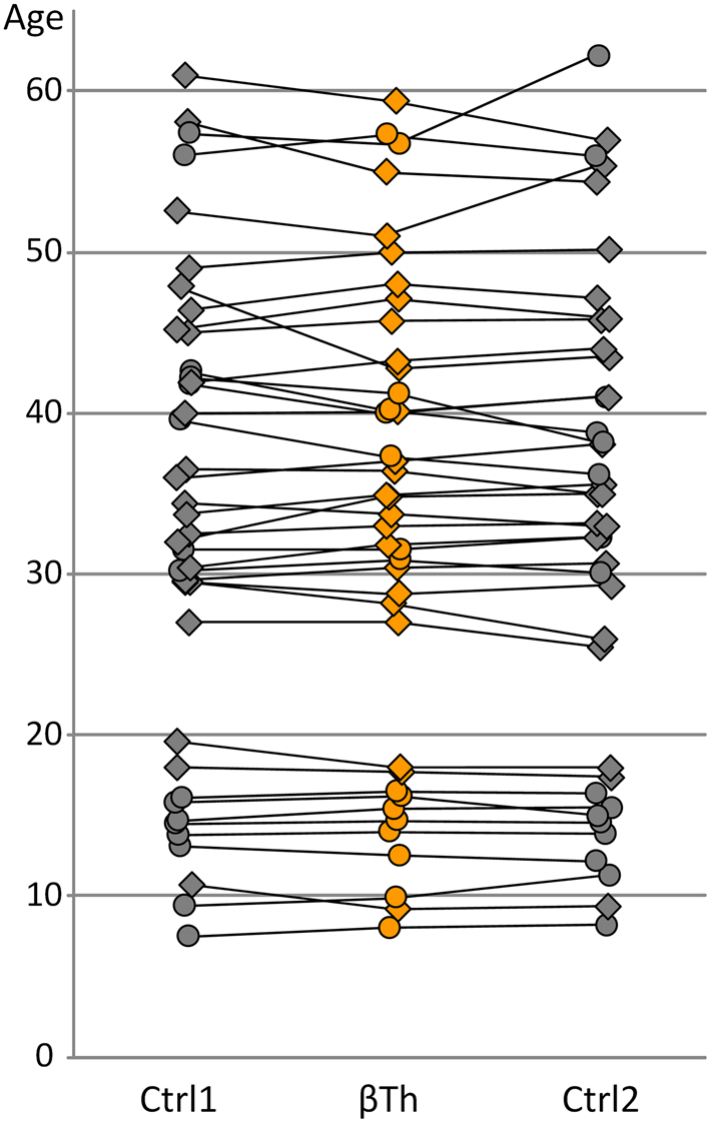
Age distribution of the βTh group (centre) and the control group (left and right). Lines connect matched subjects. Circles: females; diamonds: males.

**Figure 2:**
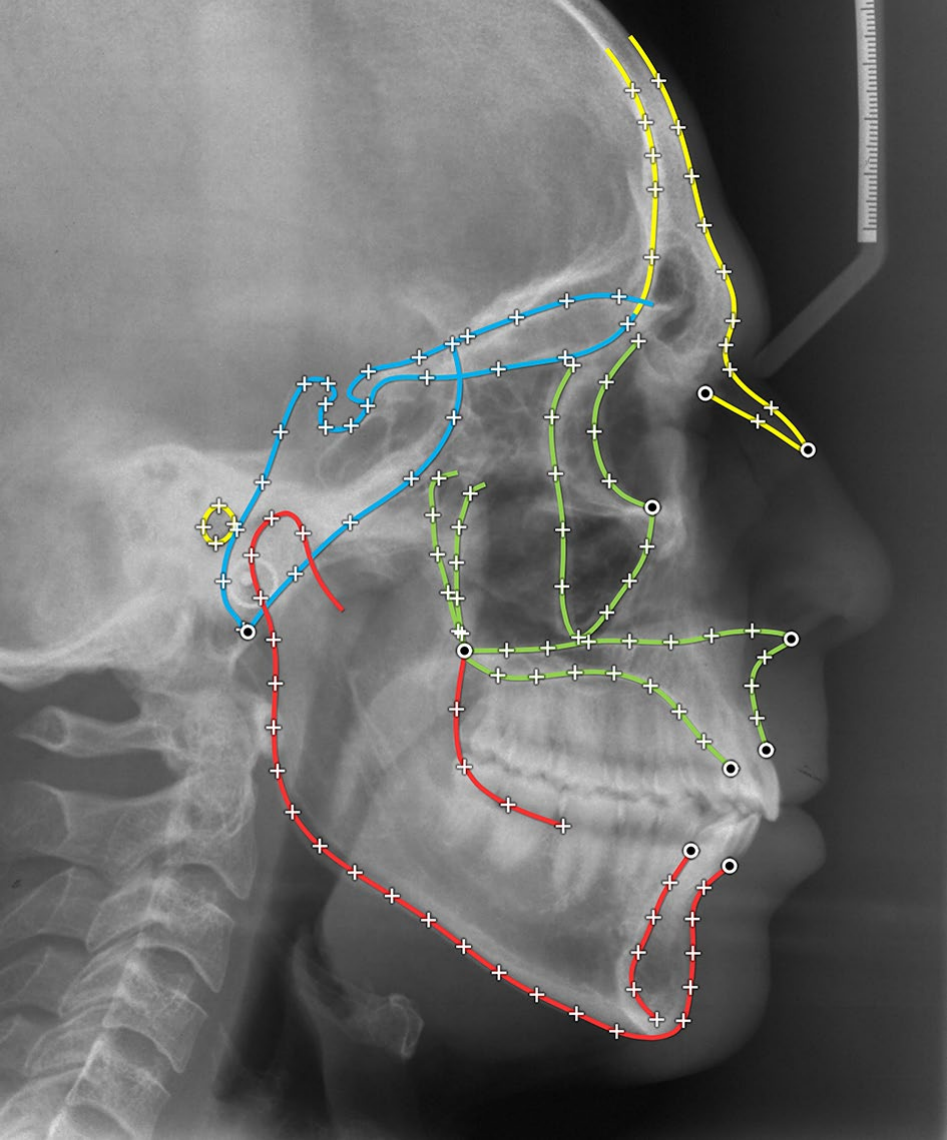
The digitized curves and landmarks. Circles: fixed landmarks; crosses: sliding semilandmarks, initially placed at equidistant positions, then allowed to slide. Blue: cranial base structures (CrB); green: maxillary structures (Mx); red: mandible (Mnd).

The present study evaluated a sample of cephalometric radiographs of patients with beta thalassaemia (βTh) and matched healthy controls (Ctrl) (Fig. 1). GM methods were applied in order to compare the shape of the craniofacial structures. Salient structures on the radiographs were digitized and described by 10 fixed landmarks and 117 sliding semilandmarks (Fig. 2). In addition to the entire craniofacial shape (CrF), we focused on three sub-regions; the cranial base (CrB), the maxilla (Mx) and the mandible (Mnd). Generalized Procrustes alignment and Principal Components Analysis (PCA) were applied to extract principal components (PCs) of shape variation.

## Results

### Overall craniofacial shape (CrF)

**Figure 3:**
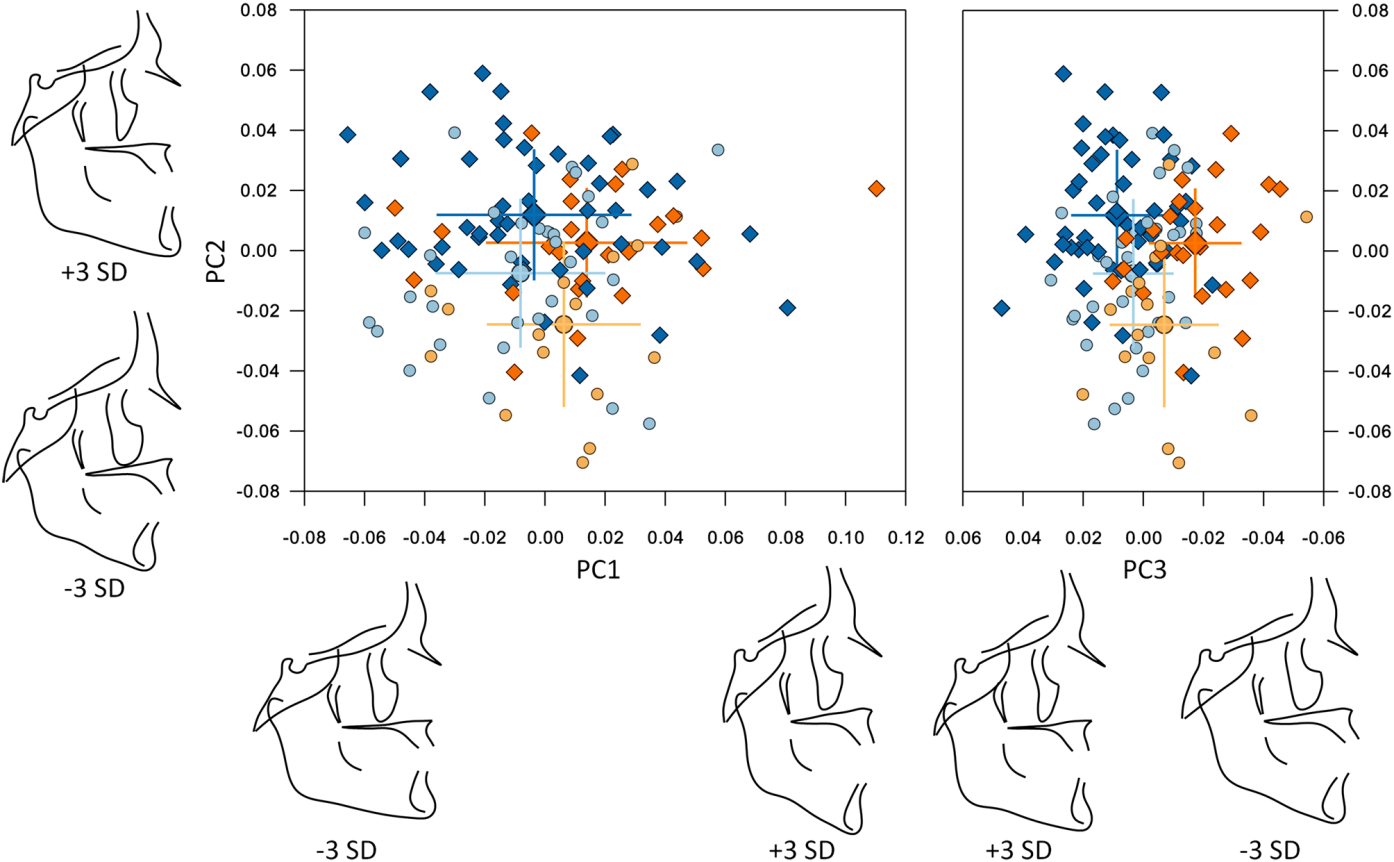
Composite of the shape space and first 3 PCs for the CrF region. Centre and right: the subjects plotted in the 3D shape space (centre: PC2 vs. PC1, right: PC2 vs. PC3). Blue and light blue: control subjects; orange and light orange: βTh group. Circles: females; diamonds: males. Large markers show group means with standard deviation lines. Axes scaling is in Procrustes distances from the average (all subjects scaled to a centroid size of 1). Left and bottom: the extreme shapes of variation along the PC1, PC2 and PC3 axes. Each extreme is + 3 or − 3 standard deviations from the average.

Approximately 83% of the shape variance of the CrF region was described by the 16 principal components that individually accounted for at least 1% of the variance (Table 1). A scree plot (Supplementary Fig. S1) confirmed that the first 3 were the most important, accounting for just over 45% of shape variance. PC1 described variability in the vertical direction, divergency between the cranial base, the maxilla and the mandible, and differences in the anterior to posterior facial height ratio. PC2 was linked mainly to the anteroposterior relationship between the maxilla and the mandible, whereas PC3 described more subtle variations, related to the protrusion of the maxilla and the symphysis, and to the gonial angle (Fig. 3). Age is correlated to the first three principal components for both groups (Supplementary Fig. S2).

**Table 1:** Sample variability for the first 19 PCs of the CrF configuration.

	%variance (%)	% cumulative variance (%)
PC 1	22.80	22.80
PC 2	15.30	38.10
PC 3	7.50	45.60
PC 4	6.60	52.20
PC 5	5.60	57.80
PC 6	4.40	62.20
PC 7	3.30	65.50
PC 8	3.00	68.40
PC 9	2.70	71.10
PC 10	2.30	73.40
PC 11	2.10	75.50
PC 12	1.80	77.30
PC 13	1.70	79.00
PC 14	1.50	80.60
PC 15	1.40	82.00
PC 16	1.30	83.30
PC 17	1.00	84.30
PC 18	1.00	85.30
PC 19	1.00	86.30

**Figure 4:**
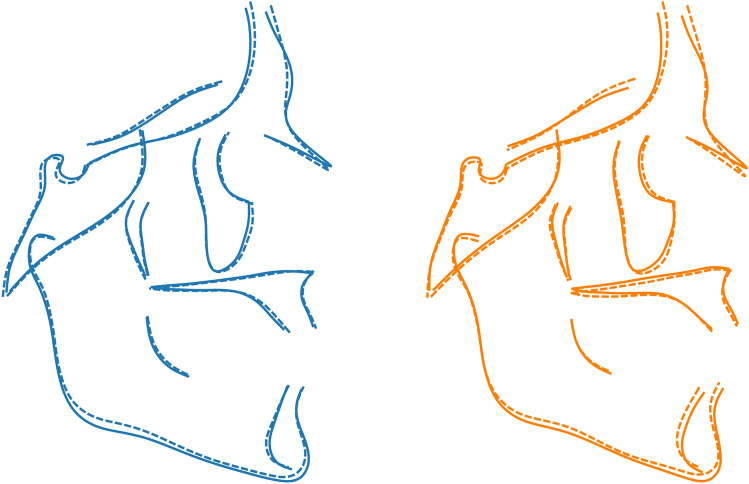
Procrustes superimposition of the average shapes of the craniofacial structures of males (solid line) and females (dotted line) of the βTh (orange) and Ctrl (blue) groups separately.

Males were significantly different than females in both the Ctrl and βTh groups (Table 2). Males had larger PC1 and PC2 values than females, signifying hyperdivergency of the skeletal pattern and tendency towards a skeletal Class III discrepancy (relative mandibular protrusion and maxillary retrusion). Differences between the sexes were mainly along the PC2 axis and were very similar in both groups, except for the PC3 axis, where the βTh males showed a tendency towards maxillary protrusion and forward inclination of the symphysis, relative to females, in contrast to the Ctrl group males (Fig. 3, Fig. 4).

**Table 2:** Permutation test results for each of the 4 regions, comparing males to females and the Ctrl group to the βTh group. All results are based on 100,000 permutations without replacement. P values are reported to the 4th decimal point.

Landmark configuration	Groups under comparison		P value (procrustes distance)	P value (Goodall’s F test)
Craniofacial (CrF)	Ctrl male	Ctrl female	0.0011	0.0012
	βTh male	βTh female	0.0037	0.0039
	Ctrl male	βTh male	0.0001	0.0001
	Ctrl female	βTh female	0.0048	0.0049
Cranial base (CrB)	Ctrl male	Ctrl female	0.0767	0.0757
	βTh male	βTh female	0.1133	0.1142
	Ctrl male	βTh male	0.1649	0.1660
	Ctrl female	βTh female	0.0968	0.1004
Maxilla (Mx)	Ctrl male	Ctrl female	0.2855	0.2851
	βTh male	βTh female	0.3505	0.3512
	Ctrl male	βTh male	0.0001	0.0000
	Ctrl female	βTh female	0.0033	0.0032
Mandible (Mnd)	Ctrl male	Ctrl female	0.0002	0.0004
	βTh male	βTh female	0.3331	0.3316
	Ctrl male	βTh male	0.0002	0.0002
	Ctrl female	βTh female	0.0056	0.0050

**Figure 5:**
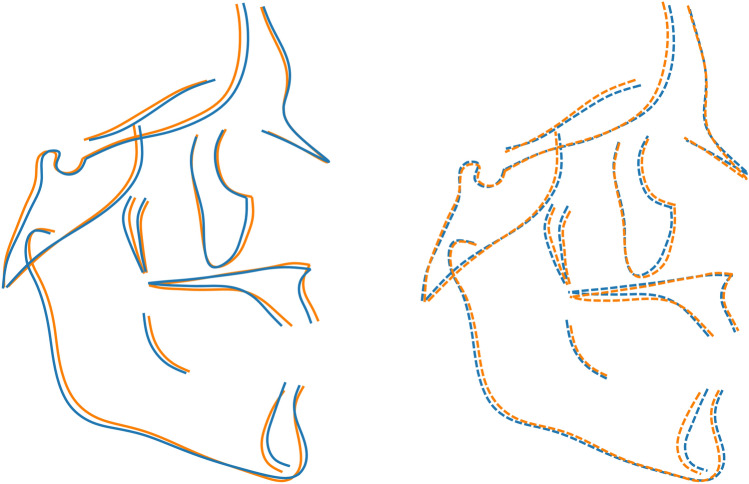
Procrustes superimposition of the average shapes of the craniofacial structures of the βTh (orange) and Ctrl (blue) groups for males (solid line) and females (dotted line) separately.

Comparison between the two groups for each sex separately showed significant differences (Fig. 3, Table 2). The βTh group showed more hyperdivergency and a Class II skeletal pattern, combined with maxillary protrusion, forward symphysis inclination and a larger gonial angle (Fig. 5).

Regression of the Procrustes coordinates on the logarithm of age showed a statistically significant correlation, but the percentage of variance explained by age was low, approximately 6%. A repeat of the inter-group comparisons, using the regression residuals, did not result in any appreciable differences to the results listed in Table 2. A scatter plot of the sample with subject markers coloured by age is shown in Supplementary Fig. 2.

### Cranial base (CrB)

Cranial shape variation showed two main patterns, aligned to PC1 and PC2, which collectively described 42.6% of shape variance (Supplementary Table S1). One pattern was related to the ratio of anterior to posterior cranial base length (PC1) and the other to the angle between the anterior and posterior cranial bases (PC2) (Supplementary Fig. S3).

**Figure 6:**
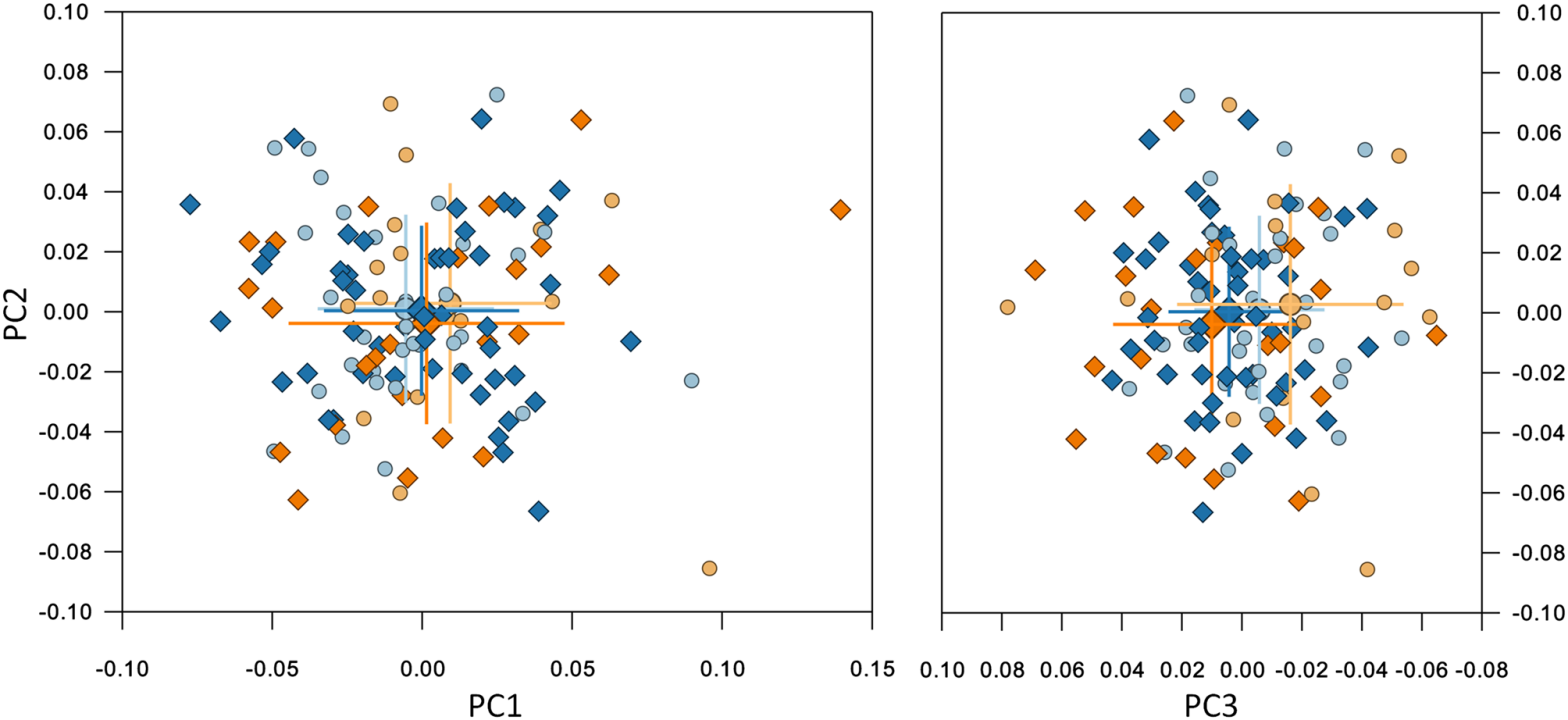
Shape space of the CrB region. Blue and light blue: control subjects; orange and light orange: βTh group. Circles: females; diamonds: males. Large markers show group means and standard deviation.

**Figure 7:**
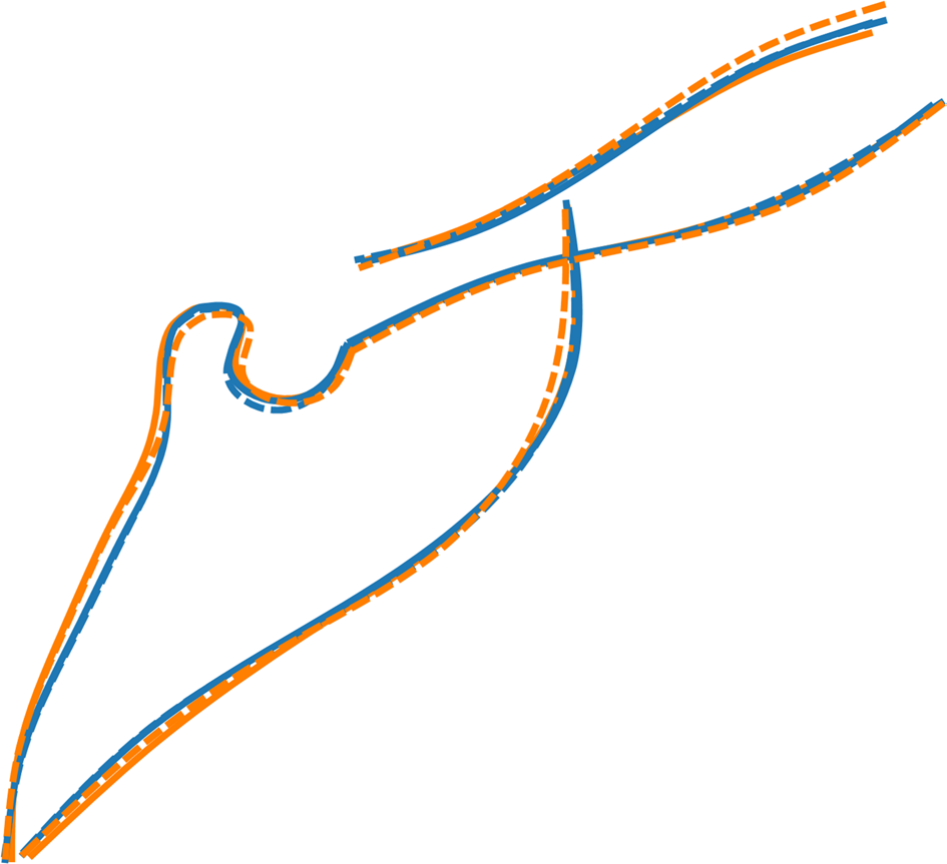
Procrustes superimposition of the average shapes of the cranial base. Orange: βTh group, blue: Ctrl group, solid line: males, dotted line: females.

No significant differences in shape of the CrB between males and females, or between the βTh and Ctrl groups, were detected (Figs. 6, 7, Table 2).

### Maxilla (Mx)

Shape variation of the maxilla was spread of several PCs, without any clear threshold in the scree plot (Supplementary Fig. S1, Supplementary Table S2). The first 3 PCs, which accounted for 50% of the shape variance, are shown in Supplementary Fig. S4.

**Figure 8:**
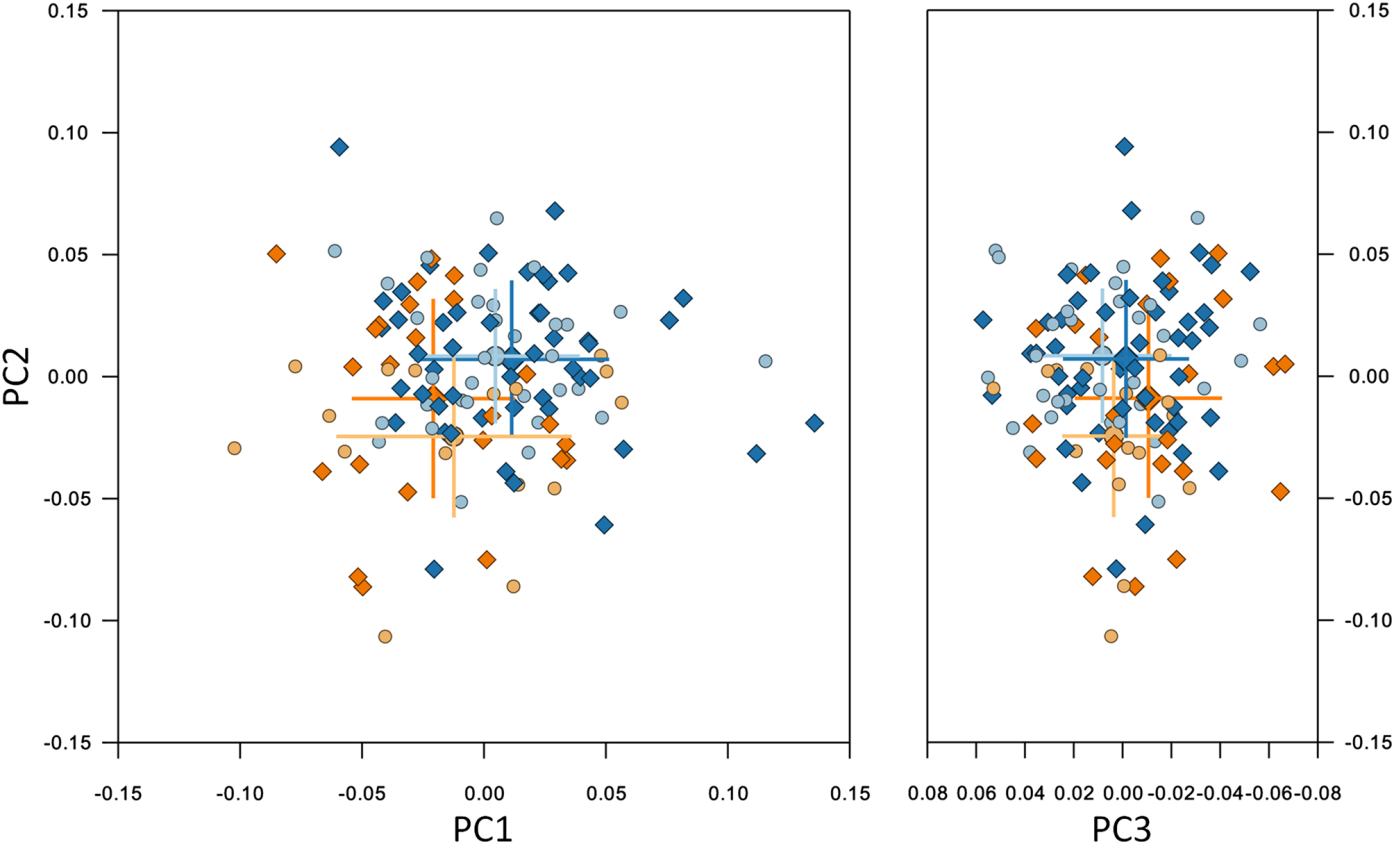
Shape space of the Mx region. Blue and light blue: control subjects; orange and light orange: βTh group. Circles: females; diamonds: males. Large markers show group means and standard deviation.

**Figure 9:**
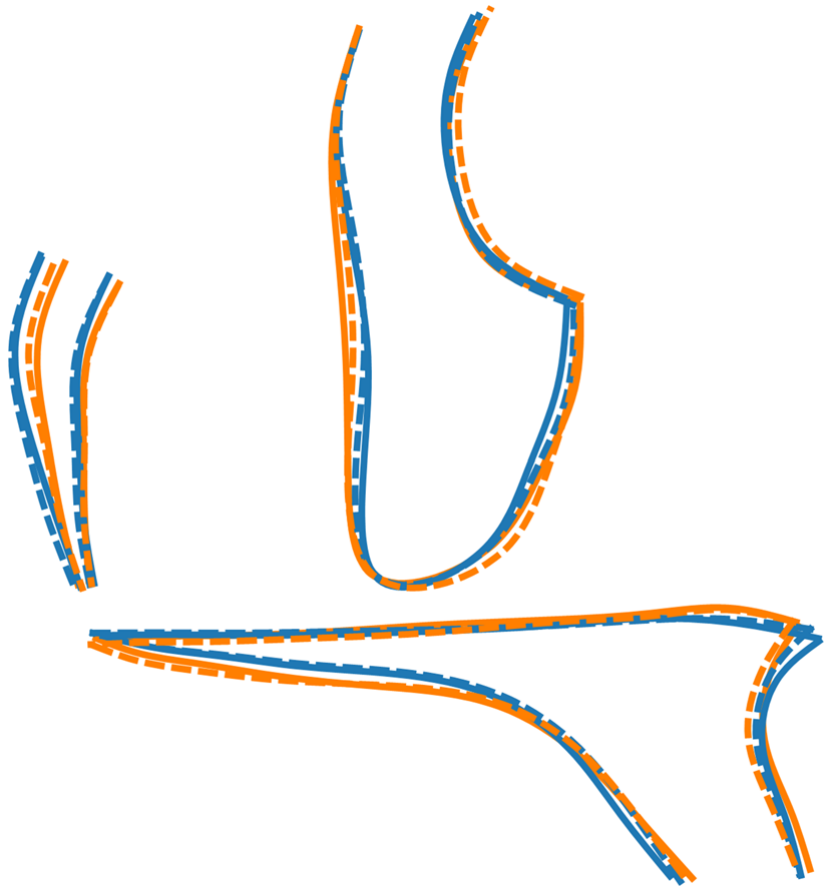
Procrustes superimposition of the average shapes of the maxilla. Orange: βTh group, blue: Ctrl group, solid line: males, dotted line: females.

Males and females were similar in shape in both groups (Fig. 8, Table 2). However, the βTh group showed significant differences to the Ctrl group, mainly in males, but also in females (Fig. 8). In the subjects with βTh, the alveolar process was protruded, the palate was thicker, the pterygomaxillary fissure was located more anteriorly, and the zygomatic process of the maxilla was wider (Fig. 9).

### Mandible (Mnd)

Shape variation of the mandible was related mainly to the gonial angle and the ratio between the length of the ramus and body. The first 3 PCs, accounting for 60% of the variance (Supplementary Fig. S5, Supplementary Table S3).

**Figure 10:**
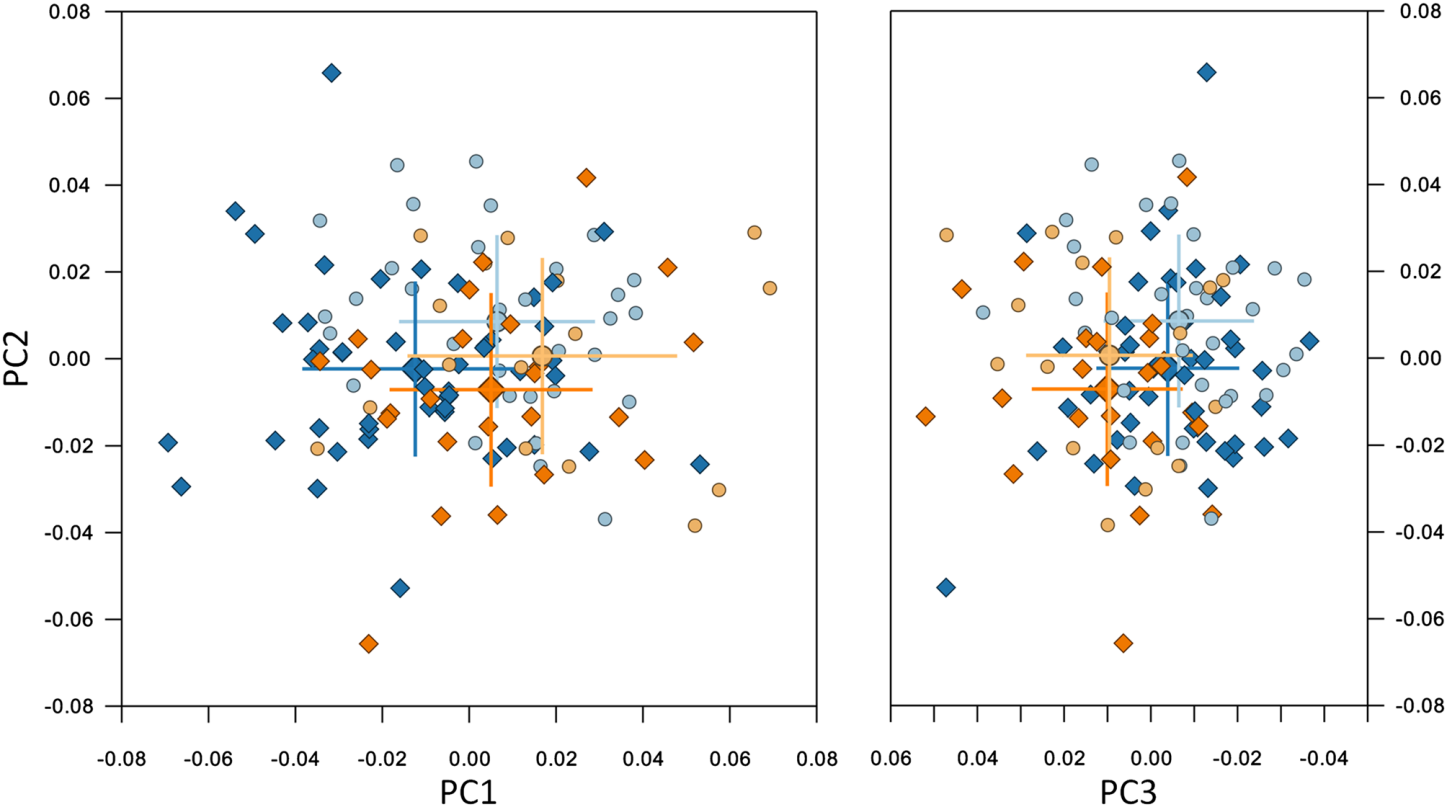
Shape space of the Mnd region. Blue and light blue: control subjects; orange and light orange: βTh group. Circles: females; diamonds: males. Large markers show group means and standard deviation.

**Figure 11:**
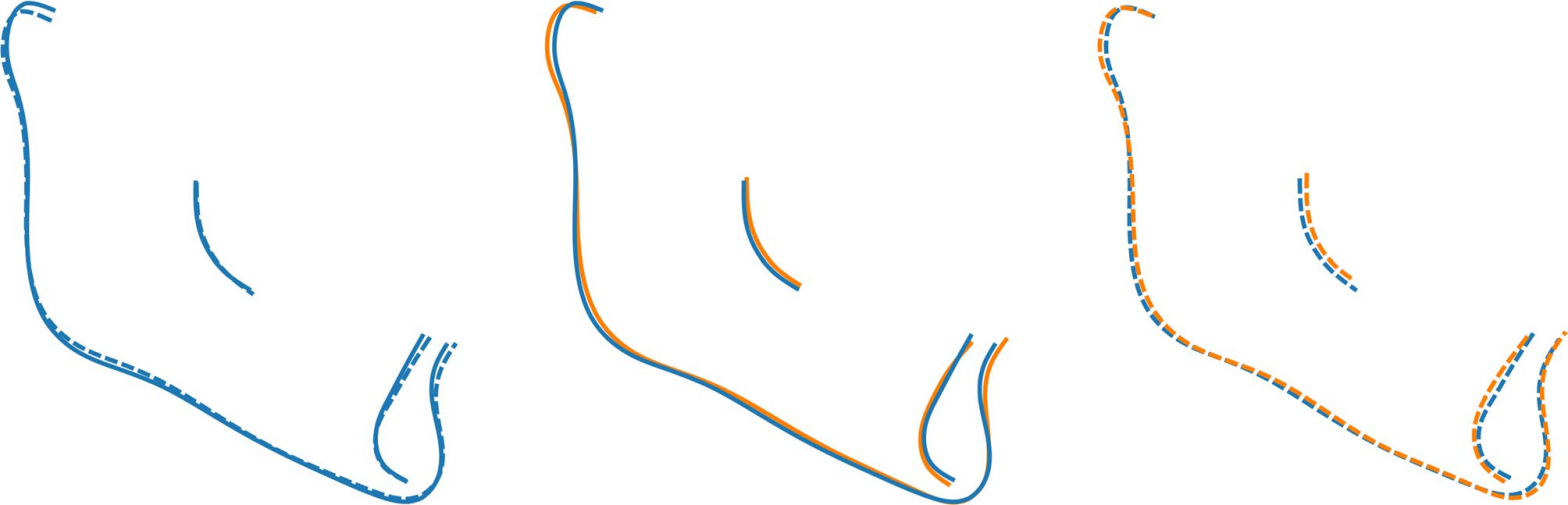
Procrustes superimposition of the average shapes of the mandible. Orange: βTh group, blue: Ctrl group, solid line: males, dotted line: females. Left: control group, centre: males of control and βTh groups, right: females of control and βTh groups.

Sexual dimorphism of mandibular shape was evident in the control group, but not in the βTh group (Fig. 10, Table 2). There was a statistical difference both between the males of the two groups, and the females. The subjects of the βTh group had a larger gonial angle, an anteriorly inclined symphysis and a larger ramal width (Fig. 11).

## Discussion

Beta thalassaemia requires multidisciplinary management; dental professionals contribute decisively to the treatment of patients with this condition. Correction of their malocclusion can be achieved with either orthodontic or combined orthodontic-surgical approach^30,31^. The orthodontic-surgical approach may be more indicated, since beta thalassaemia affects the growth of the craniofacial complex. However, heavy bleeding, stability problems due to multiple segmental osteotomies and the need to keep hemoglobin levels over 10 mg/dl postoperatively are considerations that make this approach not suitable for all patients with beta thalassaemia^30,32^. During treatment with fixed orthodontic appliances the forces are suggested to be lighter and the appointments to be more frequent because of the thinner cortical bone that may hasten tooth movement in beta thalassaemia patients^8^.

Initial observational studies of the craniofacial complex of thalassaemia patients have shown that they are more prone to a Class II skeletal pattern (increased facial convexity with the mandible positioned posteriorly at the sagittal level in relation to the maxilla), without being able to define the exact structural differences from the normal population that could support their findings^10,11^. Further observational anthropometric studies are available^14,32–36^, while 6 more studies^15–17,37–39^ and one systematic review^40^ could be found in the literature. These studies used conventional cephalometrics to compare beta thalassaemia patients with controls. This is the first study to use geometric morphometrics to evaluate the cranial structure of these patients. The results point to the same direction with the previous observational and cephalometric studies.

Toman et al. (2011)^38^ concluded that the vertical dimension is the most affected in thalassaemia patients. They demonstrated an increase in the angles related to mandibular plane inclination and a reduction in posterior face height. They also reported a shorter mandibular body and ramus, but no differences in maxillary length. These findings agree with the results of the present study. Moreover, the present morphometric approach presented a reduction of the posterior to anterior facial height ratio. The counter clockwise rotation of the maxilla contributed to this height discrepancy too.

Amini et al. (2007)^15^ agree with the results of the Toman et al. (2011)^38^ study regarding maxillary length and position in relation to the cranial base, as well as regarding the smaller and retruded mandible. Vertical differences with reduction in posterior face height and a vertical face pattern were additionally reported. Concerning the cranial base, Amini et al. (2007)^15^ did not find any differences.

Abu Alhaija et al. (2002)^16^ examined patients with beta thalassaemia major radiographically and clinically and divided the sample in three categories according to patients’ dental age. Since the differences between sexes in their study were not statistically significant, male and female measurements in each dental age group were pooled. The authors used both conventional and the Wylie analysis in order to overcome the potential error of using the Nasion point, since beta thalassaemia patients may present with depression of the nose bridge. They reported a Class II pattern because of the relative projection of the maxilla on a shorter mandible and cranial base without augmentation in maxillary length. Superimposition of the two groups in the present investigation did not find a great amount of relocation of Nasion point, nor any differences in the length of the cranial base; however it highlights the relative projection of the maxilla. Both studies agree on a hyperdivergent profile with reduced posterior facial height.

Akkurt et al. (2017)^37^ evaluated lateral cephalometric radiographs obtained from cone-beam computed tomographic images of beta thalassaemia patients. The angular measurements revealed a hyperdivergent Class II skeletal pattern (maxillary prognathism), whereas the linear measurements also indicated an increased length of the maxilla and no statistically significant differences in length of the cranial base. The dental and soft tissue variables showed protrusive lower incisors, retrusive upper incisors and an increased nasolabial angle in the beta thalassaemia sample.

As far as sexual dimorphism is concerned, conventional cephalometric studies have shown that men have increased total facial height and mandibular size, regarding both sagittal and vertical dimensions^18^, yet the mandible is located in a clockwise rotated position in women. No sexual dimorphism in maxillary prognathism or inclination has been reported^6,41,42^. Geometric morphometric analysis has shown adequate power for assessment of sexual dimorphism^43,44^. The present study agrees with the results emerged through both methodological approaches. Moreover, the present morphometric approach provided additional information on sexual differences, in that the zygomatic area was less protruded, whereas the frontal area was more protruded in males compared to females.

## Conclusions

The midface in beta thalassaemia patients was protruded with a counter-clockwise rotation of the maxilla. The mandible was smaller, posteriorly positioned and showed an increased gonial angle and clockwise rotation, resulting in a relative reduction of the posterior facial height.

The shape of the craniofacial complex in these patients is prone to be more convex and divergent.

Shape differences tend to be more pronounced in male than in female patients.

## Methods

This study was approved by the Ethics and Research Committee of the School of Dentistry, National and Kapodistrian University of Athens, Greece (147/31.5.2010). Informed consent was obtained from all participants and/or their legal guardians. All methods were carried out in accordance with relevant guidelines and regulations.

### Sample collection and inclusion criteria

The patients of the beta thalassaemia group were in treatment in the Department of Hematology of “Aghia Sofia” General Children's Hospital and referred to the Department of Oral Diagnosis & Radiology, School of Dentistry, National and Kapodistrian University of Athens for dental screening and orthodontic consultation. Patients who met the following inclusion criteria were eligible to participate in the study: (a) diagnosed with beta thalassaemia, (b) of Caucasian origin, (c) without previous orthodontic treatment and (d) already having a lateral cephalometric radiograph or requiring one for diagnosis. This beta thalassaemia group (βTh) consisted of 40 patients, 24 males, 16 females, mean age 33.4 (range 8.0–59.4 years).

A control group (Ctrl) was selected from the patients seeking orthodontic treatment at the Department of Orthodontics, School of Dentistry, National and Kapodistrian University of Athens. This group consisted of 80 healthy individuals, 48 males and 32 females, mean age 33.1 (range 7.9–60.4 years), who met the following inclusion criteria: (a) Caucasian origin, (b) no craniofacial malformations or syndromes, (c) no previous orthodontic treatment, and (d) requiring a lateral cephalometric radiograph for diagnosis and treatment planning. Each patient of the beta thalassaemia group was age and sex matched to two individuals from the control group. The median age difference between each βTh subject and their matched control was 0.8 years (interquartile range: 0.3–1.4) (Fig. 1). The cephalometric radiographs were taken with the aid of a cephalostat that ensures reproducible and stable patient positioning, aligning the mid-sagittal plane parallel to the film and perpendicular to the central ray.

### Morphometric methodology

The lateral cephalometric radiographs were scanned at a resolution of 150 dpi (Epson 1600 scanner, Seiko Epson Corporation, Nagano, Japan) and digitized using the Viewbox 4 software (dHAL software, Kifissia, Greece) by the same examiner (PR). The main craniofacial structures were described by 15 digitized curves, represented by 127 landmarks for the morphometric analysis^45^. Ten landmarks were fixed and 117 were semilandmarks, allowed to slide from their initial position along a vector tangent to their respective curve (Fig. 2).

Four landmark configurations were considered: (a) the mandible (Mnd), consisting of 3 curves and 34 landmarks, (b) the maxillary structures (Mx), 6 curves and 45 landmarks, (c) the cranial base (CrB), 4 curves and 27 landmarks, and (d) the whole craniofacial configuration (CrF), 15 curves, 127 landmarks. Independently for each of these configurations, the following procedure was followed:

1. The curves were digitized.
2. The fixed landmarks were automatically located at the end points of their respective curves.
3. The semilandmarks were automatically placed by the software at predefined equidistant positions.
4. The average of the total sample was computed.
5. The semilandmarks were slid to minimize bending energy relative to the pre-computed average of step 4. Sliding was performed along the tangent to the curve and was followed by projection of the landmark on the curve^46,47^. This step was repeated 6 times to ensure convergence.

Steps 4 and 5 were repeated 3 times, each time recomputing the new average.

Following sliding, the data were subjected to a generalized Procrustes alignment (GPA)^21^ and Principal Component Analysis (PCA), to extract the Principal Components (PCs) of shape variation. Digitization, sliding, GPA and PCA were performed with the Viewbox software.

### Statistical analysis

Groups were compared by permutation tests in the Viewbox software (100,000 permutations, with and without replacement) using the Procrustes distance between group means and Goodall’s F statistic as test criteria. All comparisons were conducted separately for each sex, and separately for each of the 4 landmarks configurations (CrB, Mx, Mnd, CrF).

To test for a potential effect of age on craniofacial shape, a regression of the shape variables on the logarithm of age was run using MorphoJ^48^. Group comparisons were repeated using the residuals of the regression.

### Error estimation

Intra-observer error was evaluated by re-tracing 20 randomly selected radiographs one month after the first tracing. Inter-observer error was assessed by tracing the same 20 radiographs by a second examiner with previous experience in digitizing cephalometric radiographs for morphometric studies. The Procrustes distance between the repeated digitizations was measured in shape space and assessed relative to the sample’s shape variation, which was computed as the diagonal across the shape space.

Intra- and inter-observer errors were computed as 9.6% and 10.9% of the total sample’s variance, respectively.

## Supplementary information

Supplementary Information 1.

Supplementary Information 2.

## Data Availability

All data generated or analysed during this study are included in this published article and its Supplementary Information files.
