# Supplementary material for: Craniofacial shape in patients with beta thalassaemia: a geometric morphometric analysis
Source: Sci Rep. 2021 Jan 18;11:1686. doi: 10.1038/s41598-020-80234-z (PMC7814138; doi:10.1038/s41598-020-80234-z)
Supplement: Supplementary file 1 — Supplementary Information 1. [file 41598_2020_80234_MOESM1_ESM.pdf]

Craniofacial shape in patients with beta thalassaemia: a geometric morphometric analysis

Petros Roussos[1], Anastasia Mitsea[2], Demetrios Halazonetis[3], Iosif Sifakakis[4*]

[1] Postgraduate student, Department of Orthodontics, School of Dentistry, National and Kapodistrian University of Athens, Greece.

[2] Assistant Professor, Department of Oral Diagnosis and Radiology, School of Dentistry, National and Kapodistrian University of Athens, Greece.

[3] Professor and Head, Department of Orthodontics, School of Dentistry, National and Kapodistrian University of Athens, Greece.

[4] Assistant Professor, Department of Orthodontics, School of Dentistry, National and Kapodistrian University of Athens, Greece.

Supplementary Information

Scree plots

Supplementary Figure S1. Scree plots of the variance explained by the first principal components of each shape configuration. The PCs that explain 1% or more of the variance are plotted.

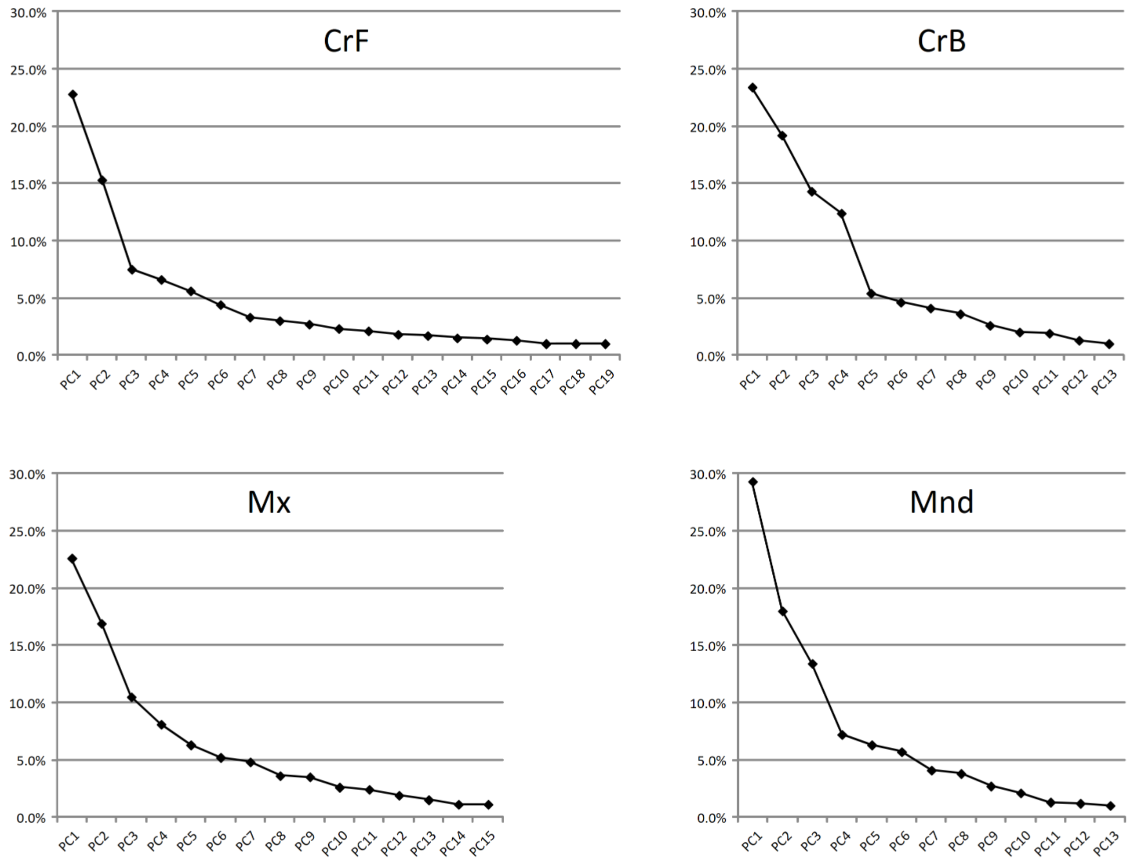

Scatter plot

Supplementary Figure S2. Scatter plot of the sample on PC1, PC2 and PC3 of the craniofacial configuration (CrF), colouring each subject by age (range: 7.5 to 62.2 years). Circles: females; diamonds: males.

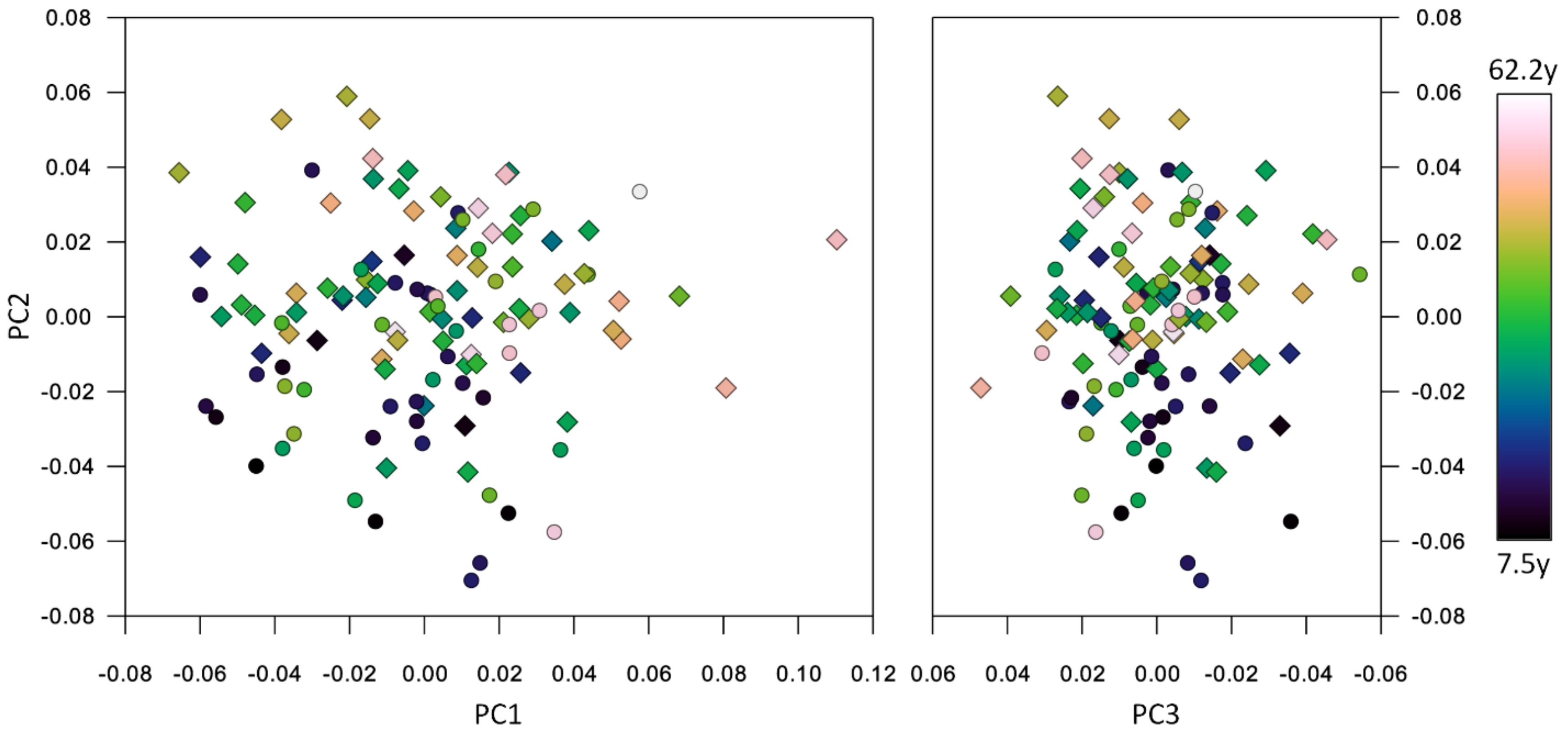

Shape plots

Supplementary Figure S3. The shape variation pattern of the cranial base, shown by extremes along PC1, PC2, PC3 and PC4. Each extreme is +3 or -3 standard deviations from the average.

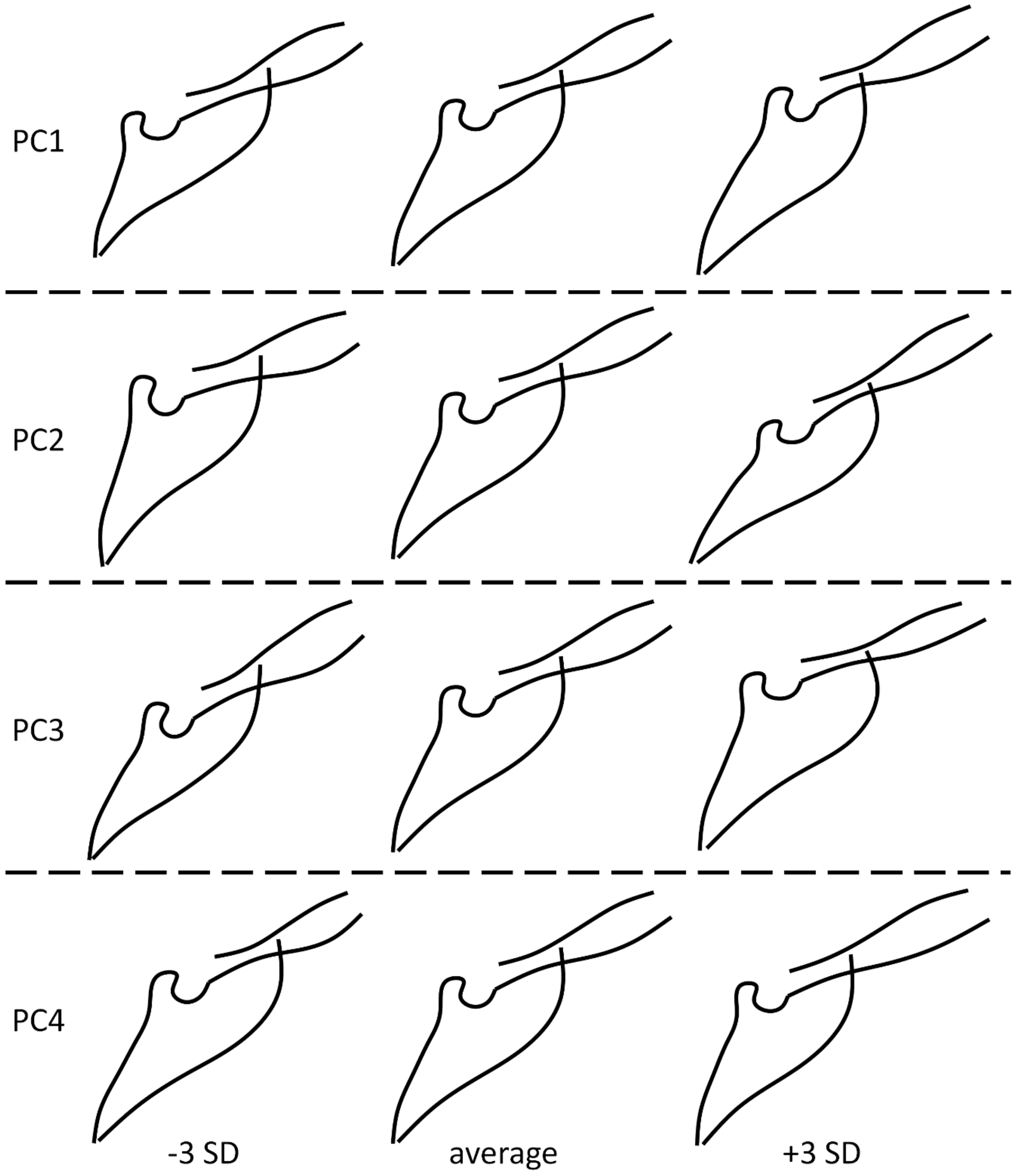

Supplementary Figure S4**.** The shape variation pattern of the maxilla, shown by extremes along PC1, PC2 and PC3. Each extreme is +3 or -3 standard deviations from the average.

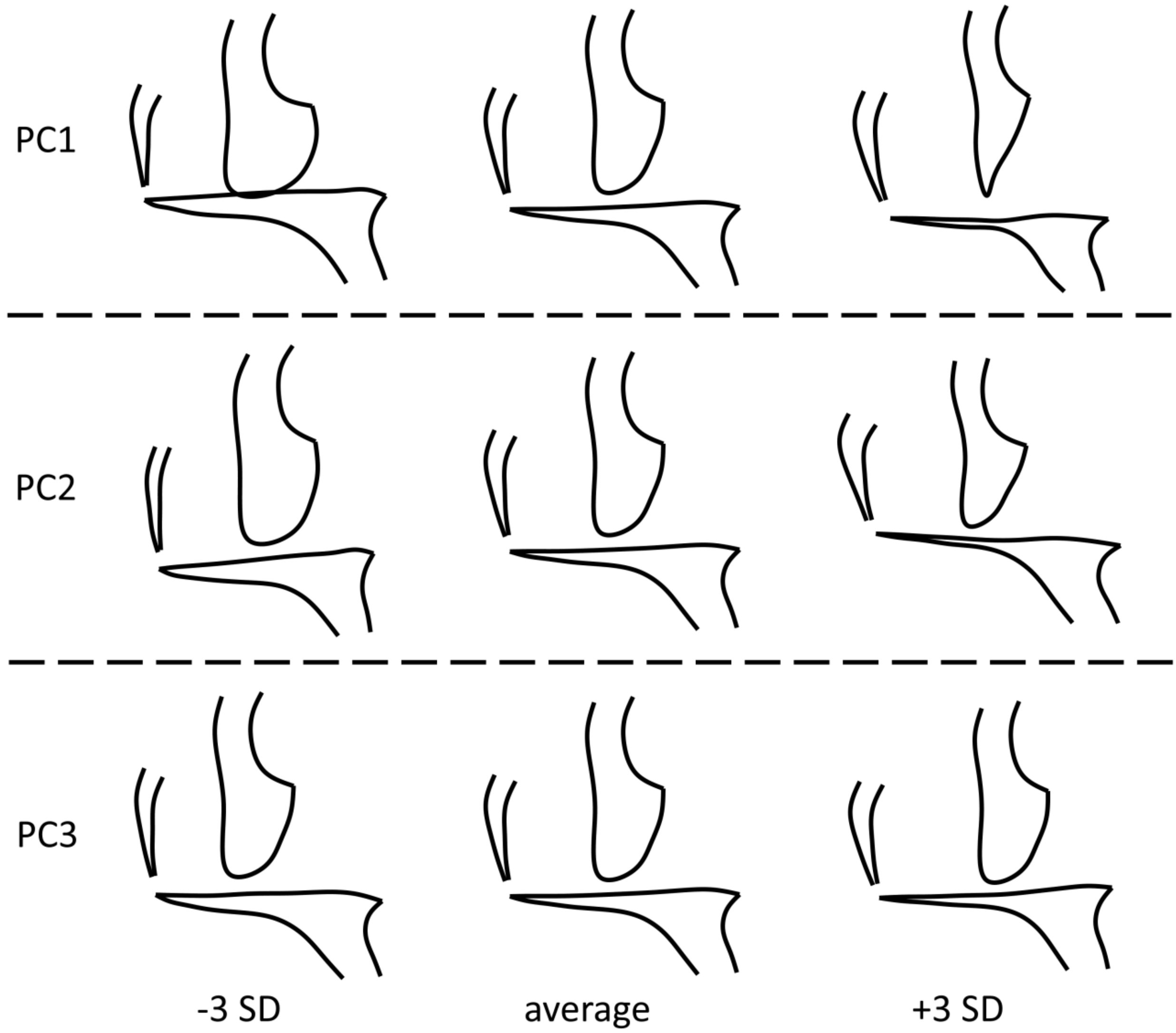

Supplementary Figure S5**.** The shape variation pattern of the mandible, shown by extremes along PC1, PC2, PC3 and PC4. Each extreme is +3 or -3 standard deviations from the average.

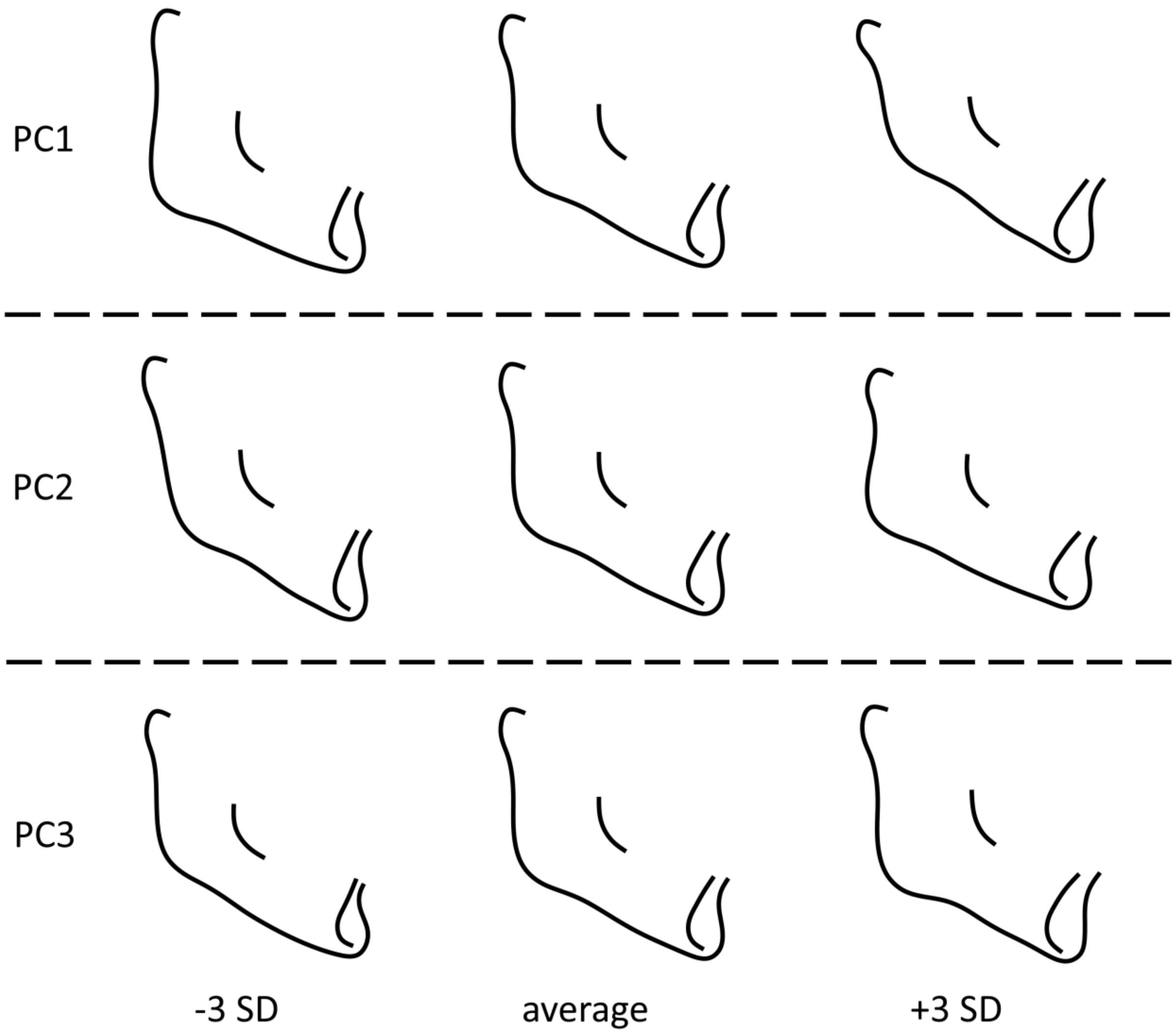

Tables

Supplementary Table S1. Sample shape variability for the first 13 PCs of the CrB configuration.

	%variance	% cumulative variance
PC 1	23.40%	23.40%
PC 2	19.20%	42.60%
PC 3	14.30%	57.00%
PC 4	12.40%	69.30%
PC 5	5.40%	74.70%
PC 6	4.60%	79.30%
PC 7	4.10%	83.40%
PC 8	3.60%	86.90%
PC 9	2.60%	89.60%
PC 10	2.00%	91.50%
PC 11	1.90%	93.40%
PC 12	1.30%	94.70%
PC 13	1.00%	95.70%

Supplementary Table S2. Sample shape variability for the first 15 PCs of the Mx configuration.

	%variance	% cumulative variance
PC 1	22.60%	22.60%
PC 2	16.90%	39.60%
PC 3	10.50%	50.10%
PC 4	8.10%	58.10%
PC 5	6.30%	64.50%
PC 6	5.20%	69.70%
PC 7	4.80%	74.50%
PC 8	3.60%	78.10%
PC 9	3.50%	81.60%
PC 10	2.60%	84.20%
PC 11	2.40%	86.60%
PC 12	1.90%	88.40%
PC 13	1.50%	89.90%
PC 14	1.10%	91.00%
PC 15	1.10%	92.20%

Supplementary Table S3. Sample shape variability for the first 13 PCs of the Mnd configuration.

	%variance	% cumulative variance
PC 1	29.30%	29.30%
PC 2	18.00%	47.30%
PC 3	13.40%	60.70%
PC 4	7.20%	68.00%
PC 5	6.30%	74.30%
PC 6	5.70%	80.00%
PC 7	4.10%	84.10%
PC 8	3.80%	87.90%
PC 9	2.70%	90.50%
PC 10	2.10%	92.60%
PC 11	1.30%	93.90%
PC 12	1.20%	95.10%
PC 13	1.00%	96.10%
